# Mindfulness Training Intervention With the Persian Version of the Mindfulness Training Mobile App for Premenstrual Syndrome: A Randomized Controlled Trial

**DOI:** 10.3389/fpsyt.2022.922360

**Published:** 2022-06-17

**Authors:** Dorsa Mazaheri Asadi, Komeil Zahedi Tajrishi, Banafsheh Gharaei

**Affiliations:** Department of Clinical Psychology, School of Behavioral Sciences and Mental Health, Tehran Institute of Psychiatry, Iran University of Medical Sciences, Tehran, Iran

**Keywords:** Mindfulness, premenstrual syndrome, quality of life, Mindfulness-app, mobile health (MHealth)

## Abstract

Premenstrual syndrome (PMS) is a common problem for women of reproductive age, affecting various aspects of their lives. While various in-person psychotherapeutic interventions, including Mindfulness therapy, have yielded promising results in reducing PMS symptoms, due to the COVID-19 Pandemic, psychotherapists are providing their services *via* online methods. Therefore, the present study is the first one worldwide to examine whether smartphone-based Mindfulness training can reduce symptoms of PMS and improve the quality of life in women with PMS. We recruited 80 Iranian women (aged 25–45) with PMS through online advertising who were randomly allocated into two groups of 40. The intervention group underwent two online introductory group sessions followed by 8 weeks of Mindfulness practice, while the control group received no intervention. Before and after the intervention, participants completed the 12-Item Short-Form Health Survey and the Premenstrual Symptoms Screening (PSST) questionnaires. Data were analyzed using the Analysis of Covariance (ANCOVA). Following the intervention, the mean scores of PMS symptoms were significantly lower in the intervention group than in the control group (*p* < 0.001; η^2^ = 0.18). Likewise, the quality of life score was significantly higher in the intervention group (*p* < 0.001; η^2^ = 0.14). Our results indicated that the smartphone-based Mindfulness training intervention could be an effective treatment modality for women with PMS symptoms, especially during the COVID-19 Pandemic, which has posed limitations for in-person therapies.

**Clinical Trial Registration:**

https://fa.irct.ir/trial/59924, identifier: IRCT20180607040000N2.

## Introduction

Premenstrual syndrome (PMS) is a cyclic phenomenon of somatic and affective symptoms arising during the days preceding menses, disturbing one's regular functioning, followed by a symptom-free interval (1). The American Psychiatric Association (APA) has provided criteria for diagnosing premenstrual dysphoric disorder (PMDD), a severe form of PMS. Accordingly, women are diagnosed with PMDD when seriously impacted by moderate to severe symptoms, as described in the Diagnostic and Statistical Manual of Mental Disorders, fifth edition (2). In another definition proposed by the American College of Obstetricians and Gynecologists (ACOG), PMS is a clinical syndrome marked by the cyclic existence of physical and emotional symptoms irrelevant to any organic disease that occur during the 5 days before menses in each of the three prior menstrual cycles and disappear within 4 days of the onset of menses, without recurrence until at least process day (3).

PMS is one of the most common and disturbing health problems among women of childbearing age (4). Recent studies have indicated that more than 75% of women experience some degree of PMS during their reproductive years (5). The prevalence of PMS has been found to be 48% worldwide and 54.9% among the Iranian community sample (6). PMS can affect all age groups; however, the most commonly affected age group includes those 25–45 years old, though the regular treatment-seeking time is the mid or late third decade of life (7–9). Depending on the course of the condition, symptoms can begin from the onset of the first menstrual period and, if left untreated, can often continue from a woman's reproductive years to menopause (10).

More than 200 mild to severe symptoms have been described for PMS, all of which interfere with the daily life of affected women (11, 12). However, the common signs that characterize this syndrome include psychological (e.g., irritability, mood swings, depression, anxiety, and impulsivity), physical (e.g., bloating, breast tenderness, pain, and headache), and behavioral (e.g., lack of coordination, sleep disorders and changes in appetite) symptoms (13, 14). PMS strongly and negatively affects women's quality of life and health, resulting in a significant decrease in mental wellbeing, productivity at work, and academic performance. It also makes the patients more likely to visit a doctor, increasing healthcare costs. Therefore, there is a need for effective treatment for PMS.

Unfortunately, treatment interventions for PMS have remained a significant challenge (15). Over the recent two decades, several studies have been conducted to examine the effectiveness of various pharmacological and non-pharmacological treatment alternatives on PMS (16, 17). While medications effectively relieve PMS syndrome, they have several side effects: nausea, sleep problems, sexual dysfunctions, headache, gastrointestinal problems, vomiting, and drowsiness (18, 19). Since the symptoms of PMS can be chronic and long-lasting, special attention should be paid to the side effects of medical interventions. Most women do not adhere to medical treatments (i.e., using medications) mainly due to side effects or contraindications. Patients often seek non-pharmacological treatment options to deal with the symptoms. Thus, other safe and effective treatment approaches should be considered in the treatment of PMS. In this regard, psychological interventions have produced promising results in the treatment of PMS (20).

The American College of Obstetricians and Gynecologists has identified non-medical interventions as a first-line treatment option for less severe PMS (3). These non-pharmacological approaches include: Lifestyle Changes, Exercise, Diet Change, Stress Management, Biofeedback, Cognitive-Behavioral Therapy, Relaxation Techniques, and Mindfulness (21–23).

Mindfulness therapy is one of the treatment modalities that have produced promising results in the treatment of PMS. The concept of Mindfulness was first examined by Buddhist traditions in philosophical terms unknown to readers nowadays. However, Mindfulness has extended swiftly in Western psychology research and practice, mainly because of the successful standardized Mindfulness-based interventions (24), including Mindfulness-based stress reduction (MBSR) (25) and Mindfulness-based cognitive therapy (MBCT) (26), which assimilate the core of Eastern Mindfulness practices into the Western cognitive-behavioral approach. In one of the most commonly cited definitions, Mindfulness is the awareness that arises through “paying attention in a particular way: on purpose, in the present moment, and non-judgmentally”. Likewise, Baer (27) has defined Mindfulness as “the non-judgmental observation of the ongoing stream of internal and external stimuli as they arise”.

Research on Mindfulness-based interventions (MBIs) has increased exponentially in the past decade. The most common Mindfulness-based interventions include Mindfulness-Based Stress Reduction (MBSR) (25) and Mindfulness-Based Cognitive Therapy (MBCT) (26). MBSR is a model of psychotherapy that combines Mindfulness techniques with stress management techniques (28). At the same time, Mindfulness-Based Cognitive Therapy (MBCT) is a multimodal intervention that integrates training in Mindfulness meditation and the cognitive theory of affective disorders (29).

Several studies have examined the effect of MBIs on PMS. For instance, Blut et al. (30) examined the impact of an 8-week Mindfulness-based intervention on 21 women aged 18–52 years with menstrual-related mood disorders (MRMDs). Results indicated a significant decrease in symptom severity for seven of the 11 (e.g., depression, anxiety, mood swings, and irritability) premenstrual symptoms, increased pain tolerance to the cold pressure, and decreased blood pressure reactivity to mental stress. Likewise, Panahi and Faramarzi (15) investigated the effect of Mindfulness-based cognitive therapy on depression and anxiety in women with PMS in a randomized controlled trial with 60 samples. The results showed that MBCT intervention is acceptable and potentially beneficial in women with PMS symptoms. Psychotherapy should be considered a treatment option for mild to moderate PMS in women with depressive symptoms. In another study, Khaleghi et al. (31) examined the effectiveness of Mindfulness training on inner happiness and non-impulsive behavior with a sample of 40 women with PMS. The results showed that Mindfulness training effectively increased inner satisfaction and reduced non-impulsive behavior in women with PMS.

In recent years, many health services have been severely affected by the COVID-19 Pandemic. Governments took decisive actions to restrict residents' freedom to reduce the contagion, forcing people to avoid unnecessary face-to-face interactions and social gatherings and limiting their movement to the strictly necessary. Like other healthcare treatments, psychotherapy was not subject to entire governmental regulations, and therapists could maintain in-person psychotherapy sessions. However, doing so was practically challenged in private clinics and public health systems, considering that face-to-face meetings could increase the risk of infection for both therapists and patients. Thus, psychotherapists were encouraged to provide their professional services *via* digital devices to guarantee the continuation of previously active therapeutic interventions. This state of affairs has sparked an unprecedented embrace of virtual healthcare technologies. Therefore, considering the prevalence of PMS and the effectiveness of psychotherapeutic interventions such as MBIs on PMS, it is necessary to develop some smartphone-based interventions to provide psychological services for this group of patients during the COVID-19 epidemic.

Virtual technologies have been widely used to provide mindfulness training (e.g., Mindfulness-based smartphone applications) (32). A meta-analysis indicated that digital Mindfulness training has significant beneficial effects on stress, anxiety, depression, and mental health (33). Likewise, several recent studies showed that Mindfulness training provided through a smartphone app reduced depressive symptoms and improved QOL and wellbeing (34). Also, Friis-Healy et al. (35) found that virtually-delivered psychotherapeutic interventions can increase therapeutic alliance for patients resistant to in-person therapies due to financial constraints or stigma.

To our knowledge, no study has examined the effect of smartphone-based Mindfulness training intervention on reducing the symptoms of PMS. Developing such treatment is necessary given the restrictions that have been raised due to the COVID-19 Pandemic. Besides, this type of intervention will benefit PMS patients who cannot attend in-person therapy because of employment, housekeeping, having a young child, living in remote areas, suffering from physical disabilities, and having heart diseases. Therefore, the present study aimed to examine the effect of smartphone-based Mindfulness training intervention on reducing PMS symptoms and increasing the quality of life (QOL) in a sample of individuals with PMS and a control group using the Persian version of the Mindfulness training mobile app.

## Methods

### Participants

**Figure 1:**
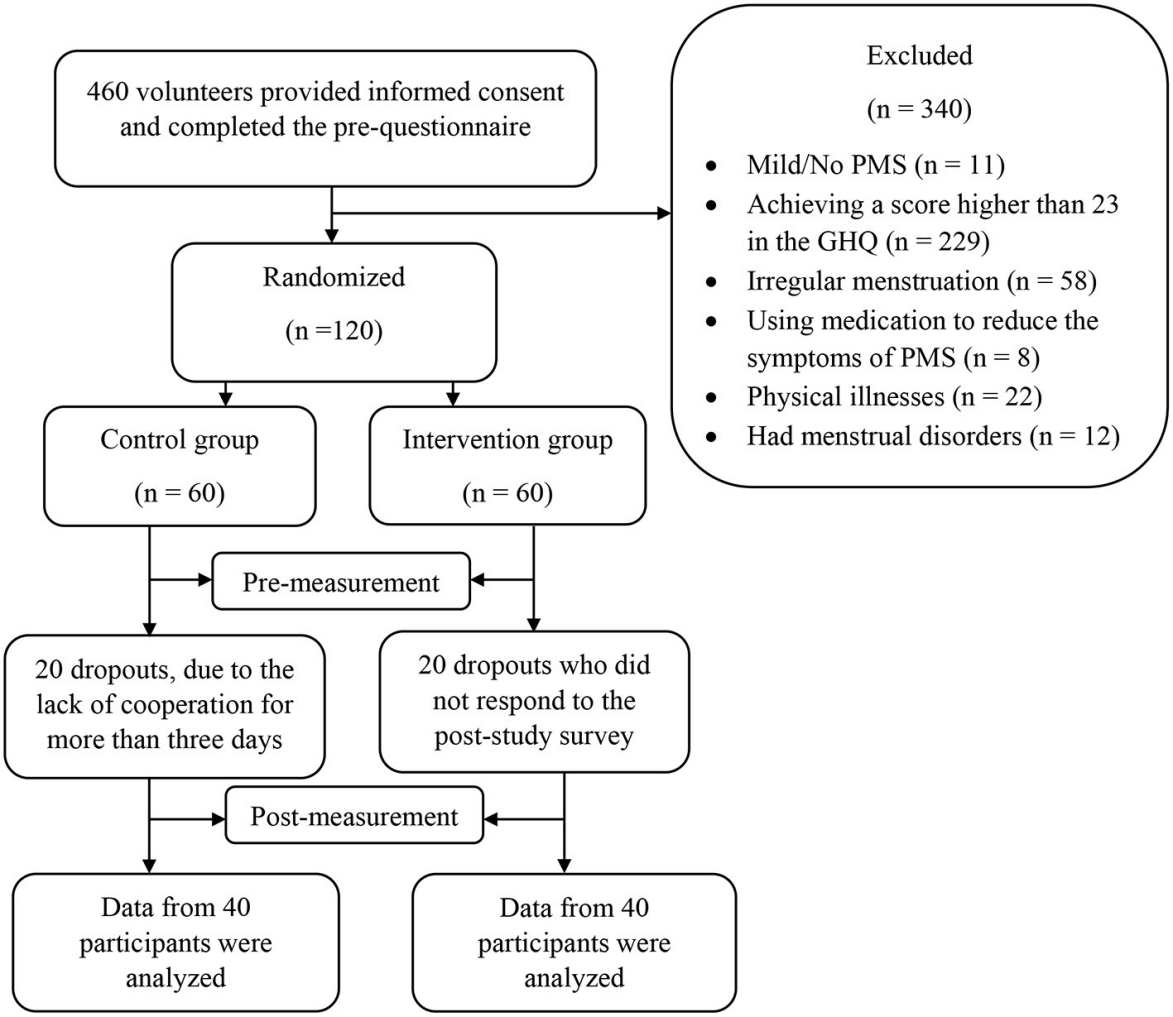
Process chart from recruitment to post-measurement.

A two-group randomized controlled, pretest-posttest design was used. Participants were recruited through online advertising through social media sites and poster advertisements. At first, 460 individuals showed interest in participating in the study and completed the pre-questionnaires after providing online signed informed consent; however, 340 individuals were excluded from the study based on the exclusion/inclusion criteria (Table 1). The G-power program was used to calculate the minimum sample size needed for our study, with an alpha of 0.05, a power of 90%, and an expected medium effect size (*r* = 0.6). It was determined that a minimum of 72 participants was required to find statistically significant differences, though considering the possibility of dropouts, we included a total of 120 individuals (60 per group) who were randomly assigned to one of the intervention and control groups based on the simple randomization method using the rand function of Excel software (A graphic depiction of the recruitment process is presented in Figure 1). Participants were selected based on exclusion/inclusion from women who completed the online pre-questionnaires shared on social media (Table 1). Of the 60 participants in the intervention group, 20 participants were dropped out before completing the 8-week intervention due to a lack of cooperation for more than 3 days. Likewise, 20 participants dropped out of the control group because they did not respond to the post-study survey. Therefore, data of 80 participants from intervention group (*n* = 40, *M* age = 31.42, *SD* = 4.82) and control group (*n* = 40, *M* age = 32.62, *SD* = 6.15) were analyzed.

**Table 1:** Inclusion and exclusion criteria.

**Inclusion criteria**	**Exclusion criteria**
1) Age range of 25–45 years old (This is because the symptoms of PMS usually start between the ages of 25 and 45) (36). 2) Having an education higher than a high school diploma.^a^ 3) Having moderate or severe premenstrual syndrome based on the Premenstrual Symptoms Screening Tool (PSST) (To confirm the diagnosis of moderate or severe PMS; all the following three conditions need to be present in the PSST together: (a) At least one moderate or severe option among the items 1–4 (first part of the PSST); (b) At least four moderate or severe options among the items 1–14 (first part of the PSST); (c) At least one moderate or severe option among the items of the second part of the PSST (37). 4) Having regular menstruation (intervals between two menstrual periods should be 33–28 days, and bleeding duration should be between 2 and 7 days (38). 5) Achieving a score ≤ 23 in the general health questionnaire (which represents mental health) (39) 6) Owning a smartphone	1) Having any menstrual disorders in the 3 months before the study 2) Suffering from physical and mental illnesses by using a self-report questionnaire and a general health questionnaire (GHQ-28) 3) Using any medication to reduce the symptoms of PMS 4) The lack of cooperation for more than 3 days in performing practice by application.

^a^*This was considered to ensure that the participants can easily read and comprehend the instructions and content of the sessions*.

### Procedure

This study was first reviewed and approved by the Research Deputy of Iran University of Medical Sciences (Code Number = IR.IUMS.REC.1400.659). The study was registered in the Iranian Registry of Clinical Trials (ID Number = IRCT20180607040000N2, Registration date: 2021-11-28). The 120 participants who met the criteria for participating in the study were asked to complete the remaining pre-questionnaire (i.e., the PSST, GHQ-28, and SF-12). Once the participants completed the measures, we created two groups on WhatsApp. The participants in the control group were told that they were on the waiting list and that their sessions would be held after 2 months. The first author developed the Mindfulness training application (Mindfulness Coach Persian Version; Tehran, Iran) based on the manual by Chaskalson (40). Then, the manual's content was translated to Farsi and reviewed by the authors. First, two 1-h group training sessions were held for the intervention group by the first author through Skype. The participants were familiarized with premenstrual syndrome, the foundations of Mindfulness, and how to use the Mindfulness coach app. Both sessions were recorded and shared on the WhatsApp group of the intervention group so that members could download the video files of the meeting if any of them missed any part of the sessions. Then, the download file of the application (an app file type designed for the Android platform) was shared with participants in the intervention group, and they were asked to install the app on their smartphones. The participants were instructed to perform the exercises specified by the app for at least 20 min per day for 8 consecutive weeks. For 8 weeks, daily notifications were sent to each participant to remind them to perform the exercises. Both groups were asked to complete the post-questionnaires at the end of week eight. Then, we provided the same two 1-h group training sessions for the control group, followed by the same intervention provided for the intervention group. As a reward for participating in the study, both groups' members were granted free 1-year membership for the Mindfulness training application.

### Intervention

First, two 1-h introductory sessions were provided for the intervention group (The content of these sessions with a brief description could be retrieved from Table 2). Next, the Mindfulness-based intervention was carried out using a smartphone app, which included 8 weeks of Mindfulness exercises aimed at alleviating PMS symptoms, including psychological (e.g., irritability, mood swings, depression, anxiety, and impulsivity), physical (e.g., bloating, breast tenderness, pain, and headache), and behavioral (e.g., lack of coordination, sleep disorders, and changes in appetite) symptoms and increase the quality of life. The intervention group performed specific exercises related to each week every day for 8 consecutive weeks (56 days). In each stage, step-by-step instructions and audio/video exercises were provided based on the two approaches of Mindfulness, including Mindfulness-based stress reduction (MBSR) and Mindfulness-based cognitive therapy (MBCT). The content of the sessions with a brief description is provided in Table 3.

**Table 2:** Two training sessions *via* Skype.

**Session**	**Content of the sessions**
First	Communicating, conceptualizing, providing explanations about premenstrual syndrome, familiarity with Mindfulness and its exercises
Second	The effectiveness of Mindfulness, its possible benefits for improving the symptoms of premenstrual syndrome, how to use the application

**Table 3:** Eight weeks of exercise using the Mindfulness training app.

**Week**	**Content**
First	Mindfulness eating exercise (audio)/your experience of mindful eating/body scan exercise (audio)/your experience of body scan/first week lesson/exercise at home
Second	Mindful breathing/meditation postures (visual)/awareness of breathing audio exercise/remembering and forgetting/the four important skills/second week's lesson/exercise at home
Third	Mindfulness of body movements/exercise up to the endurance threshold point/mindful stretching exercises (audio and visual)/audio breathing exercise in three steps/physical barometer/approaching and avoidance/exercise at home
Forth	Managing reactions/learning to respond rather than react/mindful walking (audio)/what is anxiety?/Mindfulness of breathing and body (audio)/Mindfulness of sounds and thoughts (audio)/inevitable awareness (audio)/activity with attention window/exercise at home
Fifth	Accept all the experiences/resilience/sitting with unfavorable feelings (audio)/be kind to yourself/breathing exercise in three steps/exercise at home
Sixth	Making sense/our thoughts are affected by our moods and feelings/Mindfulness and mental production/mindful connection with thoughts/Mindfulness accompanied by thoughts (audio)/three strategies to cope with distress/useless mental patterns/using breathing exercises to cope with thoughts/exercise at home/take good care of yourself/using the breathing exercise and choosing a practical step/act mindfully/anxiety indices and practical strategies/energizing and weakening activities/exercise at home
Seventh	Living with Mindfulness/remain mindful/21 ways to remain mindful at work/keep doing Mindfulness exercises
Eight	Living with Mindfulness/staying mindful/21 ways to stay mindful at work/keep Mindfulness exercises.

### Measures

#### General Health Questionnaire

The GHQ-28 is a self-report measure designed by Goldberg and Hillier (41) and widely used as a screening tool for mental disorders. It includes four subscales of somatic symptoms, anxiety and sleep disturbance, social dysfunction, and depression (42). Twenty-eight items of the measure are rated on a four-point Likert scoring system (from 0 to 3), with a total maximum score of 84 and a minimum of zero. Psychiatric morbidity is defined as a total score of ≥23, and a score of ≥7 in each subscale is defined as psychiatric morbidity. Studies on the validation of the GHQ-28 have demonstrated its high validity and reliability in different countries (41). The psychometrics of the Persian version of the GHQ28 was supported by Nazif et al. (39). In the current study, the reliability of GHQ-28 based on Chronbach's alpha was 0.85 for the GHQ-28 total score and ranged from 0.65 to 0.74 for the subscales scores.

#### Premenstrual Symptoms Screening Tool

PSST is a self-report questionnaire developed by Steiner et al. (43). PSST has 19-items loading on two domains. The first domain includes 14 items assessing psychological, physical, and behavioral symptoms; the second domain has five items and evaluates the impact of symptoms on women's functioning. Each item is rated on a four-point Lickert type scale (not at all = 0, mild = 1, moderate = 2, severe = 3). Hariri et al. (37) translated and validated the Persian version of the PSST. In the current study, Chronbach's alpha was 0.89, 0.82, and 0.85 for the PSST total score and subscales of mood symptoms and behavioral and physical symptoms, respectively.

#### 12-Item Short-Form Health Survey

The 12-Item Short-Form Health Survey (SF-12) is a widely used measure to evaluate health-related quality of life (HRQoL). It is a subset of the larger SF-36 and monitors health in general and specific populations. The SF-12 was designed by Ware et al. (44) and measures eight health aspects, including physical functioning, role limitations due to physical health problems, bodily pain, general health, vitality (energy/fatigue), social functioning, role limitations due to emotional issues, and mental health (psychological distress and psychological wellbeing). Two subscales are derived from the SF-12, including the Physical Component Summary (PCS) and the Mental Component Summary (MCS). Montazeri et al. (45) examined the psychometrics of the SF-12 in Iran and concluded that the measure is suitable for use with Iranian samples. Scores of 12–24 indicate poor quality of life, 25–36 indicate average quality of life, and 48–37 show good quality of life. In the current study, Chronbach's alpha was 0.79, 0.72, and 0.74 for the SF-12 total score and subscales of mental and physical health, respectively.

### Data Analyses

We used SPSS 20 software for data entry and statistical analyses. The normality of the distribution for outcome measures was tested using the Kolmogorov–Smirnov test, and the results supported the normality of the data (*p* > 0.05). The independent-samples *t*-test was used to detect initial differences between groups in age. At the same time, the Chi-square test was implemented to compare descriptive variables of the groups, including marital status and level of education. We performed a series of analyses of covariance (ANCOVA) to determine whether the groups differed in their reported levels of behavioral and somatic symptoms of PMS and reported levels of QOL scores using baseline values as covariates. The following rules of thumb are used to interpret values for Partial eta squared: η^2^ = 0.01 indicates a small effect; η^2^ = 0.06 indicates a medium effect; η^2^ = 0.14 indicates a large effect. It was decided beforehand that a *p* level of <0.05 would be accepted as indicating statistically significant results.

## Results

As shown in Tables 4, 5, groups did not differ significantly on demographic variables, including marital status, level of education, and age. Thus, the groups were matched in these variables. To examine if the Mindfulness intervention has resulted in significant differences in PSST and SF-12 total scores (dependent variables), two sets of ANCOVAs with baseline values as covariates were conducted. Results indicated that the intervention group scored significantly lower than the control group in total score of PSST [*F*_(1, 75)_ = 2.27, *p* < 0.001, η^2^ = 0.18] and higher in terms of the SF-12 total score [*F*_(1, 75)_ = 68.65, *p* < 0.001, η^2^ = 0.14]. Further, to examine whether the groups scored significantly different in the subscales scores of PSST and SF-12, we conducted four one-way ANCOVAs, with- baseline values as covariates. Results revealed that the intervention group had significantly lower PSST mood [Wilks' λ = 0.275; *F*_(1, 75)_ = 17.20; *p* < 0.001; η^2^ = 0.17] and behavioral/physical symptoms scores [Wilks' λ = 0.275; *F*_(1, 75)_ = 89.21; *p* < 0.001; η^2^ = 0.15] than the control group. Similarly, the intervention group scored significantly higher in SF-12 physical health [Wilks' λ = 0.331; *F*_(1, 75)_ = 5.50; *p* < 0.001; η^2^ = 0.11] and mental health [Wilks' λ = 0.275; *F*_(1, 75)_ = 11.15; *p* < 0.001; η^2^ = 0.15] scores compared with the control group. Table 6 presents the means and standard deviations of study variables in pre-test and post-test for each group.

**Table 4:** The comparison of age between control and intervention groups.

**Variable**	**Group**		**Mean comparison**	
	**Intervention**	**Control**	***t***	***p***
	**Mean (*SD*)**	**Mean (*SD*)**	
Age	31.42 (4.89)	32.62 (6.15)	−0.996	0.337

*SD, Standard deviation*.

**Table 5:** The comparison of demographic data between control and intervention groups.

**Variables**	**Groups**			**Comparison**	
	**Intervention (*n* = 40)**	**Control (*n* = 40)**	**Total (*n* = 80)**	***X* ^2^**	***p***
**Marital status (%)**
Married	12 (30)	16 (40)	28 (35)	0.905	0.636
Unmarried	26 (65)	22 (55)	48 (60)		
Divorced/widowed	2 (5)	2 (5)	4 (5)		
**Education (%)**
Diploma	3 (7.5)	6 (15)	13 (11.3)	3.850	0.278
Bachelor	14 (35)	19 (47.5)	33 (41.3)		
Master	18 (45)	13 (32.5)	27 (38.8)		
Doctoral	5 (12.5)	2 (5)	7 (8.8)		

**Table 6:** Descriptive information about the premenstrual symptoms, quality of life scores in pre-test and post-test.

**Variable**	**Group**	**Pre-test**	**Post-test**
		**Mean (*SD*)**	**Mean (*SD*)**
**PSST total score**	Intervention	27.59 (4.77)	18.09 (4.47)
	Control	22.57 (3.40)	26.27 (4.76)
Mood symptoms	Intervention	11.50 (2.29)	6.37 (2.17)
	Control	9.72 (1.82)	10.35 (1.75)
Behavioral and physical symptoms	Intervention	16.45 (3.75)	11.72 (3.89)
	Control	12.85 (3.49)	15.92 (3.54)
**SF-12 total score**	Intervention	28.52 (4.28)	36.95 (3.81)
	Control	35.97 (4.78)	31.02 (5.31)
Physical health	Intervention	14.07 (1.92)	17.62 (1.73)
	Control	15.80 (2.20)	15.72 (2.26)
Mental health	Intervention	14.45 (2.97)	19.35 (2.74)
	Control	15.50 (2.18)	15.30 (1.93)

*SD, Standard deviation; PSST, Premenstrual Symptoms Screening Tool; SF-12, 12-Item Short-Form Health Survey*.

## Discussion

This study aimed to examine the effectiveness of smartphone-based Mindfulness training intervention in reducing PMS symptoms and increasing the quality of life (QOL) using the Persian version of the Mindfulness training mobile app. Based on the effect size values, these differences were in the strong range for the PMS symptoms and were in the moderate to strong ranges for the QOL scores assessed by SF-12. To the best of our knowledge, very few studies have examined the effectiveness of Mindfulness-based intervention on PMS symptoms. Moreover, our study is the first one worldwide to explore the efficacy of smartphone-based Mindfulness training intervention on PMS symptoms and the quality of life (QOL). Overall, our results indicated that the intervention significantly reduced the PMS' psychological, physical, and behavioral symptoms and also considerably improved the quality of life among PMS patients. We discuss our findings below.

Our findings supported the effectiveness of Mindfulness training using the Persian version of the Mindfulness training app in reducing the physical and behavioral symptoms of PMS, which is in line with a few previous research (46–48). For example, Askari et al. (46) examined the effect of MCBT on PMS symptoms; their results showed that MCBT reduced physical and behavioral symptoms of PMS significantly. Mindfulness is a balanced sense of consciousness that helps- individuals observe and accept emotions and physical experiences as clearly as they occur. Thus, in the case of PMS, Mindfulness intervention can help patients feel and accept their feelings/emotions and physical symptoms such as pain. Consequently, the extent of attention and oversensitivity to reporting these symptoms are reduced (49). Furthermore, Mindfulness helps patients be fully aware of their thoughts and feelings and accept them without judgment, be in a state of calm and concentration, and gain the ability to control their thoughts, anxiety, and emotions. As a result, Mindfulness therapy helps patients develop a remarkable ability to cope with the physical and behavioral symptoms of PMS (50). Mindfulness works exactly like when people consciously turn their awareness to the experiences they are going through here and now. Therefore, Mindfulness treatment could have helped the participants become aware of their cognitive, behavioral, and emotional responses to PMS symptoms, allowing patients to modify their relationship with their painful thoughts and feelings on PMS symptoms and reduce the impact of these thoughts and feelings on their lives (51).

Furthermore, our results indicated a significant reduction in mood symptoms of PMS after the Mindfulness treatment, a finding consistent with previous studies (15, 30, 48). For example, Bluth et al. (30) examined the effect of an 8-week Mindfulness-based intervention on 21 women with menstrually related mood disorders (MRMDs). Results indicated a significant reduction in symptom severity for seven of the 11 (e.g., depression, anxiety, mood swings, and irritability) PMS symptoms. Similarly, Panahi and Faramarzi (15) explored the effect of MBCT on depression and anxiety symptoms in PMS patients, and the results showed that MBCT significantly reduced PMS symptoms and anxiety and depression symptoms. In this regard, it could be said that Mindfulness encourages combining meditation and specific mental orientations toward an experience and awareness of the present in a non-judgmental way by minimizing the conflict in thoughts and feelings. Mindfulness meditation activates an area of the brain that creates positive emotions, has beneficial effects on the body's immune function, and alleviates depression (52). Following Mindfulness training, the person learns to identify stressful situations, become fully aware of them, and consciously control experiences such as depression, anxiety, and stress. As soon as the individuals master their reactions in-depth, they can modify their responses to stressful, anxiety-provoking, and depressive situations and use positive instead of negative ones (53). Given its non-judgmental- approach to internal experiences (feelings and cognitions), Mindfulness allows individuals to reduce their automatic reactions in dealing with stressful experiences. With the passage of time and increased awareness and acceptance of life incidents that can be changed, activation of response systems to stress and physical symptoms decreases (15).

Finally, our findings showed the significant effectiveness of Mindfulness training in improving the physical and mental dimensions of the quality of life in individuals with PMS, which dovetails with the results of prior studies (54–56). For example, van Emmerik et al. (54) examined the effect of Mindfulness-Based Mobile Application intervention on quality of life, general psychiatric symptoms, and self-actualization. The results showed that it is possible to achieve the long-lasting positive impact of Mindfulness intervention on general psychiatric symptoms and several aspects of quality of life at low costs with smartphone apps for Mindfulness. Mindfulness can improve the quality of life in several ways: First, Mindfulness helps individuals notice that cognition, thoughts, and feelings are fleeting thoughts and feelings. This attitude reduces rumination, and automatic thoughts, leading to reduced negative emotions and unpleasant reactions and effective management of these emotions (57). Second, while Mindfulness emphasizes the acceptance of the current situation, individuals with PMS try to change the actual unfavorable situation. Thus, Mindfulness focuses on helping patients be satisfied with the current situation rather than sustained efforts to address the possible future issues. Accepting the recent situation results in an elevated sense of- satisfaction and happiness, which is not conditional on the type of experience. Finally, Mindfulness may improve women's physical and psychological health with PMS by encouraging them to perform muscle relaxation techniques and reducing aggravating physical reactions to stress, resulting in improved quality of life (58).

The results from the current study should be interpreted considering a few limitations. First, due to the lack of access to the intervention group after the post-treatment assessment, we could not perform a follow-up analysis to examine the long-term effects of the intervention. It is suggested that future research include follow-ups to examine the long-term effectiveness of the intervention. Second, we did not use a Mindfulness measure to see if the Mindfulness scores had significantly enhanced after the intervention. Third, using a sample of volunteers is also a limitation, as there may be a reason to self-select, which leads to bias. Fourth, using self-report measures for primary outcomes may be considered a limitation, though this is how PMS is commonly measured. Fifth, analyses in this study followed a per-protocol design but not an intention-to-treat one; the latter type of study design is used to nullify the effects of crossover and dropout, which may break the random assignment to the treatment groups in a study. Sixth, our study sample included highly educated women suffering from moderate to severe PMS; thus, the results should be interpreted only for individuals with high education. Finally, since this study was the first worldwide to examine smartphone-based Mindfulness intervention with a PMS sample, we suggest future studies replicate the results in various cultures. This includes translating the app to other languages and examining the efficacy of the intervention.

## Conclusion and Clinical Implications

Our findings suggested that smartphone-based Mindfulness training intervention is an effective treatment for highly educated individuals suffering from moderate to severe PMS. More specifically, this intervention could be an excellent treatment modality during the COVID-19 Pandemic, wherein therapists prefer online therapies over in-person interventions because of the safety of both parties. Such interventions can increase access to treatment and reduce treatment costs so more patients can benefit from it.

## Data Availability Statement

The raw data supporting the conclusions of this article will be made available by the authors, without undue reservation.

## Ethics Statement

The studies involving human participants were reviewed and approved by Research Deputy of Iran University of Medical Sciences (Code Number: IR.IUMS.REC.1400.659). The patients/participants provided their written informed consent to participate in this study.

## Author Contributions

DM designed the Mindfulness coach app, performed the intervention, and prepared the manuscript. KZ and BG supervised the study and reviewed and revised the manuscript. All authors have contributed to the study and agreed to the publication of the manuscript. All authors contributed to the article and approved the submitted version.

## Funding

This study was funded by the Iran University of Medical Sciences (IUMS), Tehran, Iran; Grant Number = IUMS-1400-1-50-20718.

## Conflict of Interest

The authors declare that the research was conducted in the absence of any commercial or financial relationships that could be construed as a potential conflict of interest.

## Publisher's Note

All claims expressed in this article are solely those of the authors and do not necessarily represent those of their affiliated organizations, or those of the publisher, the editors and the reviewers. Any product that may be evaluated in this article, or claim that may be made by its manufacturer, is not guaranteed or endorsed by the publisher.
